# Atlanta Society of Medicine

**Published:** 1894-03

**Authors:** R. R. Kime

**Affiliations:** Secretary; 63½ Whitehall Street


### ATLANTA SOCIETY OF MEDICINE.

FEBRUARY 6, 1894.

*President Dr. J. B. Duncan in the Chair.*

### REPORT OF CASES.

DR. CROWE reported a case of nervous reflex which almost simulates epilepsy. Mrs. B., white, age, —— married, was referred to my clinic at the Atlantic Polyclinic Hospital from the section on nervous diseases, to examine and give an opinion as to whether any uterine trouble existed which would act as a cause of the extreme nervous condition as presented by the patient. She gave a history dating back four years, of a nervous condition that came on gradually and continued to grow worse notwithstanding she had been treated by a number of physicians for nervous troubles, until now she had gotten so unbalanced that she could not go on the street alone. Her face was one of extreme depression, with almost every muscle of the body in a state of agitation, very much as we see in marked cases of paralysis agitans. Under any excitement she would loose her balance and fall into a convulsive fit very much like epilepsy. On examination I found the following condition present: Uterus and appendages normal, but about three-quarters of an inch above the cervix, high up at the vault of the vagina, was a constricted band that looked very much as if you had ligated the cervix; this constriction almost completely closed the cervical canal, and looked like a pedunculated fibroid the size of an English walnut protruding from the cervix. Under a treatment of complete dilatation of cervix with general tonics and bromides, she has improved very fast and promises a fair recovery.

DR. COOPER: *Case* 1.—Had operated upon an unusual case a short time since at Grady Hospital. A negro man brought in who claimed to have been kicked in abdomen. I was not called until next afternoon; found him with a violent septic peritonitis; temperature 102°; at —— P. M. called Dr. Elkin, and at —— P. M. temperature 96°, pulse ——. The temperature at —— A. M., taken by house surgeon, was ——; at —— P. M. incisionunder chloroform; intestines all united together; one quart or more of pus, fecal matter, etc., escaped from incision; no bruising or ecchymosis of skin or abdominal wall at sight of injury; adhesions everywhere; while separating them fecal matter, pus and lymph escaped in abundance; after a difficult search found a rupture of small intestine completely torn across to mesentery, which was trimmed and approximated by the Czerny-Lembert suture. After breaking all adhesions we could find and flushing abdomen with twenty gallons of warm water closed incision. Operation lasted two hours, used stimulants, patient rallied, regained consciousness, but sank a few hours later and died. Never saw or heard of a case where rupture was transverse and complete before this one.

*Case* 2.—Rare—operated on at Grady Hospital. Had La Grippe and what was supposed to be an irreducible hernia, and so diagnosed it, but on closer investigation found it be a tumor in right side of scrotum and inguinal canal, hard and nodular in character; pushed up scrotum and found nothing but cord in ring. Diagnosed fatty tumor, which proved correct on operating. It was very difficult to remove, had to be dissected out, blood supply very free.

DR. HARDON: I know of no case like the rupture reported by Dr. Cooper; the case is unique. I saw a case to-day, a woman thirty-five years, married to a man of seventy years. Hymen all present, but from coition had been torn off from two-thirds of its circumference, the opening in hymen annular and just large enough to admit end of index finder.

DR. CHAMPION reported a case of recurrent appendicitis. R. B. R., male, age twenty years, had a mild attack of appendicitis in May, 1893. On September the 14th, of same year, had a chill at 5 A. M., followed by a rise of temperature to 103°. At 6 P. M. had another chill, with rise of temperature to 105°. There was tenderness in right iliac region, but no decided pain until three days after commencement of attack. The pain was not very severe; only necessary to give morphine twice. On the 18th I called Dr. J. C. Avary in consultation, and we decided not to operate, thinking possibly it would pass off as the other attack.

Quinine and antipyrine were tried to control temperature, but had to resort to sponging and cold-pack. The temperature was remittent in type, and for two weeks rose to 103° or 104° in the afternoon, after which it was subnormal in the morning, with a rise to 99½° in the afternoon, quinine being given in large doses during this time. For three weeks and a half temperature did not rise above 99½°. No pain and very little soreness in abdomen, bowels regular, appetite good, pulse 90.

Six weeks after attack patient sat up several hours and temperature went up to 100 and pulse 115. On October 31st he was operated on by Dr. Avery with the assistance of Drs. Crowe, Kime, Asher and myself. The incision was four inches in length and midway between a line drawn from right anterior superior spine of ilium to umbilicus. In hunting for the appendix the ileum for about twelve inches was found to contain a number of constricting bands from a line to half an inch in width. These bands almost occluded the intestine, but it was found by considerable pressure gas could be forced through the constriction. The appendix was found, tied off and removed. It contained a hard fecal concretion, was inflamed and inflated with gas. Not being prepared to do an exsection of the bowel, patient having been under the anesthetic for some time, and believing, or rather hoping, the manipulation with the bowel and nature would remedy the trouble, the abdomen was closed up. The patient reacted well from the operation and was kept on liquid diet for nine weeks, when he commenced to take some solids and now feels as well as usual and takes all the solid food he wants. I believe the cause of both attacks was the fecal concretion, the inflammatory process extending into the cecum and ileum and producing the constriction.

Dr. Wyeth, in his text-book on surgery, says bands of cicatricial tissue resulting from peritonitis cause intestinal obstruction in a certain proportion of cases. This accident occurs chiefly in adults, about equally in both sexes, being due to pelvic inflammations in women, and to typhlitis and traumatic peritonitis in men. Removing the cause of the trouble, breaking up the adhesion of the intestines and the gradual dilatations of the stricture by the food taken has made a complete cure.

DR. COOPER then reported case of a young friend on whom he operated. A young man subject to bilious attacks ate fried corn for supper; that night suffered such severe pain called in a doctor. I saw him next day and diagnosed appendicitis. Later called Dr. Elkin, who saw him on Saturday, Sunday and Monday, when he was so much better Dr. Elkin discontinued his visits. Temperature of patient last two days had not been above 99° F. We regarded case convalescent. Wednesday patient grew worse, saw him late in the evening and stayed over night; temperature gradually rose from 100° to 102° F.; pulse, 100; vomited two or three times during night. At day, Thursday, with Dr. Elkin, operated with a rapidly failing pulse. Incision into pus cavity, appendix free in it and perforated; general peritonitis, patient sank and died. Believed earlier operation would have saved patient. Thought relapse due to rupture of abscess. Believes in early operations.

DR. TAFT, of St. Joseph, Missouri (by invitation), had seen many cases; operated on some; believed nine-tenths of cases would get well without operation. Not a convert entirely to new pathology, but believes we have cases of typhlitis and peri-typhlitis as well.

Advises putting patient to bed, giving slight purge, danger of perforation from too free catharsis before adhesions. Believes in operating where there is sudden perforation of appendix before adhesions, rupture of abscess, or where abscess has distended walls so as to reach abscess without entering abdominal cavity.

In most cases advises absolute rest, moderate purgation with calomel and salines, later opiates. Does not believe in grape seed, corn, etc., causing appendicitis, but due to invasion of bacilli from intestines.

R. R. KIME, M. D., Secretary.

63½ *Whitehall Street.*

---

### THE SAME OLD STORY.

John Jones, an educated and successful physician, was injured in a railroad accident, and was a nervous invalid, using spirits continuously, and to excess at intervals. He came from a neurotic family, and had used spirits occasionally from early life. From the time of the accident he became more and more addicted to the use of spirits. Finally he came under my care, suffering from delusions, insomnia and general prostration. He recovered slowly, and retained ideas of persecution, believing his brother (who brought him to the asylum) wished to destroy his practice and reputation. He was impatient of restraint after the first month, and was intriguing and bitterly slanderous. After three months' treatment he went away restored, and resumed practice. The next year he relapsed, and became impulsive and reckless in his use of spirits, and was intoxicated all the time. He lost all pride of character, and associated with the lowest people. The Gold Cure Specific attracted his attention, and after three weeks' treatment he became the most enthusiastic believer in its merits. Later he was in charge of a branch asylum, then he became a lecturer, and wrote papers advocating the value of this secret specific in the most positive manner, posing as an example, and confirming his assertions by statistics that were at least startling. Asylums for inebriates who did not use Gold Cure Specifics were severely condemned, and this *Journal* was regarded as unworthy of notice. In some medical societies he obtained a hearing and made some converts, and was considered authority of great weight. Finally he disappeared, and was found in Ward's Island, New York, where he had been sent for drunkenness for thirty days. At the expiration of his sentence he drank to great excess and was found dead in a barn, probably from cerebral hemorrhage.—*Quarterly Journal of Inebriety.*

